# Dysentery and leg ulcer as an atypical presentation of systemic lupus erythematosus: A case report

**DOI:** 10.1097/MD.0000000000032201

**Published:** 2022-12-16

**Authors:** Biki Kumar Sah, Shipra Chaudhary, Ashhrik Pahari, Aasha Ghimire, Rajan Kumar Sah, Abhishek Kumar Sah, Neelam Kumari, Yaswant Kumar Jaiswal, Vivek Kumar Sah

**Affiliations:** a Department of Pediatrics and Adolescent Medicine, B.P. Koirala Institute of Health Sciences, Dharan, Nepal; b Kathmandu Medical College, Sinamangal, Kathmandu, Nepal; c Manipal College of Medical Sciences, Pokhara, Nepal; d B.P. Koirala Institute of Health Sciences, Dharan, Nepal; e Universal College of Medical Sciences and Teaching Hospital, Bhairahawa, Nepal.

**Keywords:** case report, dysentery, leg ulcer, SLE

## Abstract

Due to heterogeneity in the organs involved and a variety of influencing factors, a wide range of clinical manifestations are possible in systemic lupus erythematosus (SLE). In our knowledge, a combination of leg ulcer and dysentery as presenting symptoms of SLE has never been reported previously.

**Patient concerns:**

A 13-year-old female child presented with a chronic wound over right medial malleolus for 6 months, and passing of watery stool, later mixed with blood, for 4 days. On examination, she had a fever of 38.5°C. Lab reports revealed anemia, thrombocytopenia, proteinuria, and features of urinary tract infection. Renal biopsy showed membranous glomerulonephropathy. She was positive for antinuclear antibodies (ANA) and antidouble stranded DNA (anti-dsDNA). Immunofluorescence revealed reduced C4 and C3 levels. Abdominal ultrasound showed symmetrical circumscribed thickening, and edematous cecum and ascending colon.

**Diagnosis:**

The patient was diagnosed with SLE based on the Systemic Lupus International Collaborating Clinics classification criteria.

**Interventions:**

The patient was treated with prednisolone, hydroxychloroquine, metronidazole, ciprofloxacin, trypsin-chymotrypsin, zinc, calcium, and calcitriol tablets.

**Outcomes:**

Fever subsided within 3 days of treatment. Gastrointestinal symptoms subsided within 1 week of treatment. On 31 day of treatment, the wound had been reduced and showed features of healing.

**Conclusion:**

Dysentery and leg ulcers can be the manifestations of SLE. Therefore, SLE should also be considered when a patient presents with such symptoms. Any suspicion of infection in SLE should be treated aggressively with antibiotics.

## 1. Introduction

Systemic lupus erythematosus (SLE) is a systemic chronic inflammatory disease which is characterized by autoantibodies against self-antigens which results in inflammation-mediated damage to multiorgan. Its prevalence is 3.3 to 8.8 per 100,000 in children and adolescents.^[1]^ Studies show its incidence is more common in female children belonging to Asia and Africa with female to male ratio: 4.7 to 6.2.^[2]^

SLE generally presents with the common symptoms like fever, fatigue, myalgia, cutaneous (malar rash, photosensitivity, discoid rash), mucosal (painless oral and nasal ulcers) lesions. Some patients predominantly show manifestations in musculoskeletal, renal, nervous, respiratory, hematological, or cardiovascular system. The most frequent symptoms involving digestive system in SLE are abdominal pain, gastrointestinal infections with hepatic pathologies. The presentation of dysentery is rare in SLE. Although the incidence of oral ulcers in SLE patients is approximately 54.3%^[3]^ whereas ankle ulcers are rare with prevalence only 0.05% to 0.15%.^[4]^ The combined presentation of dysentery and ankle ulcer is even more rare. The heterogenous presentation among the patients is the reason for the delayed diagnosis. Here, we report a rare case of a patient who presented with dysentery and leg ulcer as atypical manifestations of SLE.

## 2. Case report

A 13-year-old Indo-Aryan female child born of nonconsanguineous marriage, fully immunized was brought to pediatric outpatient department with a complaint of wound over right medial malleolus since 2 months. It was initiated by a wood prick. The wound progressed gradually for which oral medication (cefixime) and wound debridement were done 2 months along with follow-up in 2 to 3 days for dressing but the wound did not subside.

Child also complained of passes of loose stool 4 days back which was acute in onset, watery in consistency, 5 to 7 episodes throughout the day and was mixed with undigested food particles. The passing of loose stool was associated with epigastric pain which was acute in onset, non-radiating, mild in severity with no aggravating and relieving factors. Following next day, she started passing blood mixed stool which was acute on onset, occurring 5 to 6 episodes a day, foul smelling, consisting of water and blood. Fever of 38.5°C was documented at the time of admission in the pediatric ward which was not associated with chills or rigor. There was no history of vomiting, abdominal distension, jaundice, fatigue, malaise, loss of consciousness, abnormal body movement, cough, shortness of breath, and difficulty in breathing. There was no family history of blood disorder, malignancy, autoimmune diseases, and other chronic diseases.

**Figure 1.**
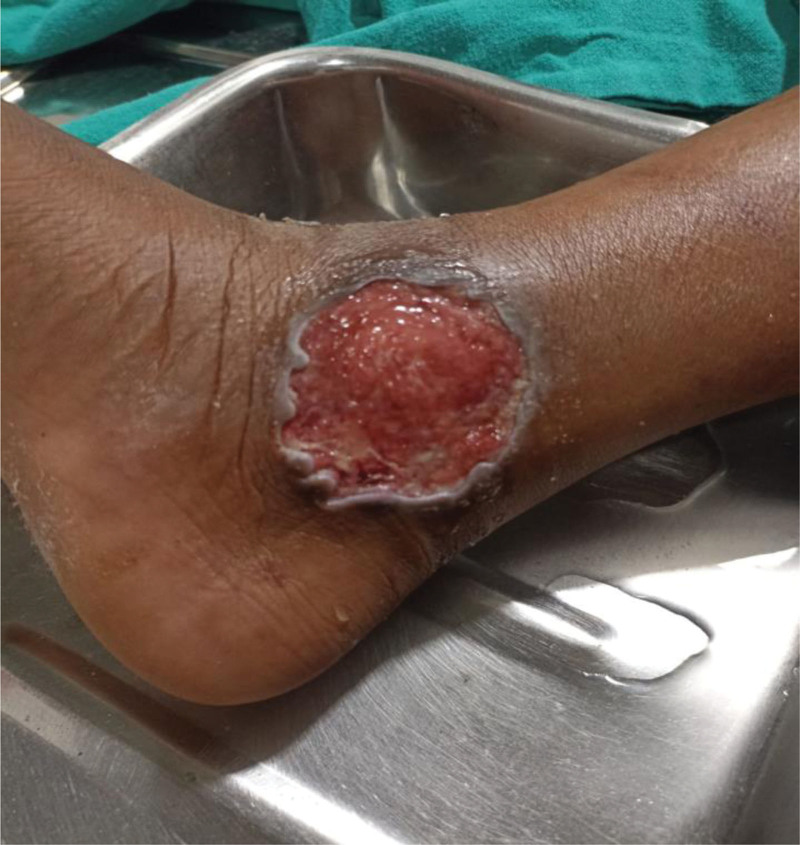
There is a single round ulcer over right medial malleolus of size approximately 6 × 6 cm punched-out borders, raised edge and base covered by red granulations tissue with minimal discharge.

On examination, the child was conscious, well oriented to time, place and person with a Glasgow Coma Scale score of 15/15. Child was thin-built with BMI between −3 and −2 SD. Vitals signs were normal. There was mild pallor with no icterus, cyanosis, clubbing, and lymphadenopathy. Child looked hydrated. Her developmental milestones were normal. On Local examination, there was presence of a single superficial ulcer over right medial malleolus, tender with size approximately 6 × 6 cm round ulcer with punched-out borders, raised edge, and base covered by red granulations tissue with minimal discharge(Fig. 1). Mild edema was present on the skin surrounding the ulcer with painful range of movement. Femoral and pedal pulses were present bilaterally. Neurologic examination was unremarkable. Systemic examinations were within normal limits.

Laboratory analysis was notable for anemia of chronic disease. Hemoglobin, 6 gm/dL (reference range, 11–16); packed cell volume, 20.2% (reference range, 35–54); mean corpuscular volume, 76.8 ft (reference range, 80–100). There was decreased serum iron, 30.62 mg/dL (reference range, 33–193); increased serum ferritin, 564.91 mg/L (reference range, 15–150), and slightly raised total iron binding capacity, 564.19 mg/dL (reference range, 135–392). RBCs showed mild anisopoikilocytosis, normocytes, microcytes with normochromic to mild hypochromia. There was also a decrease in platelet count, 131,000 cells/mm (reference range 150,00-450,000) but erythrocyte sedimentation rate persistently above 53mm/hr (reference range 0–12). There is an increased urine protein creatinine ratio, 1.99 mg/mg creatinine (reference range < 0.20) with normal urea, 21.9 (reference range 10–50) and low creatinine, 0.2 (reference range 0.3–1.2). Electrolytes and liver function tests were within normal limits.

**Figure 2.**
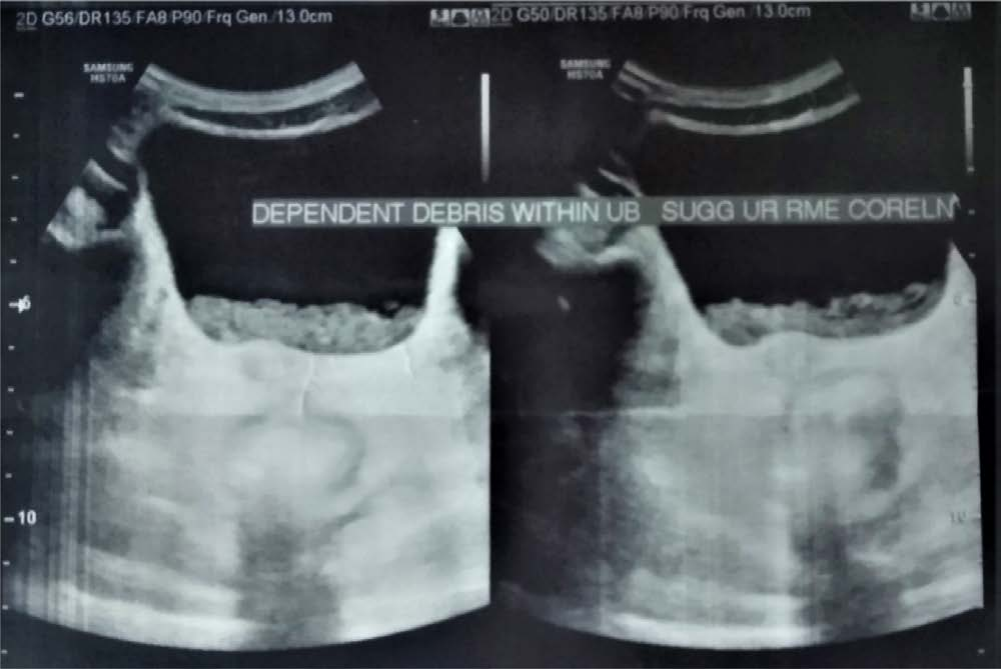
Dependent debris noted on lumen with normal distension and wall thickness of urinary bladder.

Urine routine examination and microscopy showed presence of white blood cells and bacteria and with no red blood cells and proteinuria. Other urinary test results showed 24-hour urinary protein of 0.23g/24 hours (reference range < 0.15), no malignant cells in cytology. On ultrasound of urinary bladder, dependent debris was noted on lumen with normal distension and wall thickness (Fig. 2). A stool occult blood test was positive. Stool specimen revealed the presence of *Entamoeba histolytica.* Later, renal biopsy showed membranous glomerulonephropathy, class V according to WHO classification.^[5]^

Specific tests ANA and antidouble stranded DNA (anti-dsDNA) were positive with titer > 400 AU/mL (reference range negative:<40, positive:>40) and > 800 IU/mL (reference range negative:<30, positive:>30), respectively. Immunofluorescence revealed that was decreased in C4, 0.08 mg/dL (reference range, 10-40) and C3 level, 20.65 (reference range, 90–180). Other tests performed which were negative include HIV antibody tests, hepatitis B surface antigen, and hepatitis C antigen. *Mycobacterium tuberculosis* (MTB) was not detected in Gene Xpert MTB and acid-fast bacillus was not seen in Zeihl–Neelsen stain.

**Figure 3.**
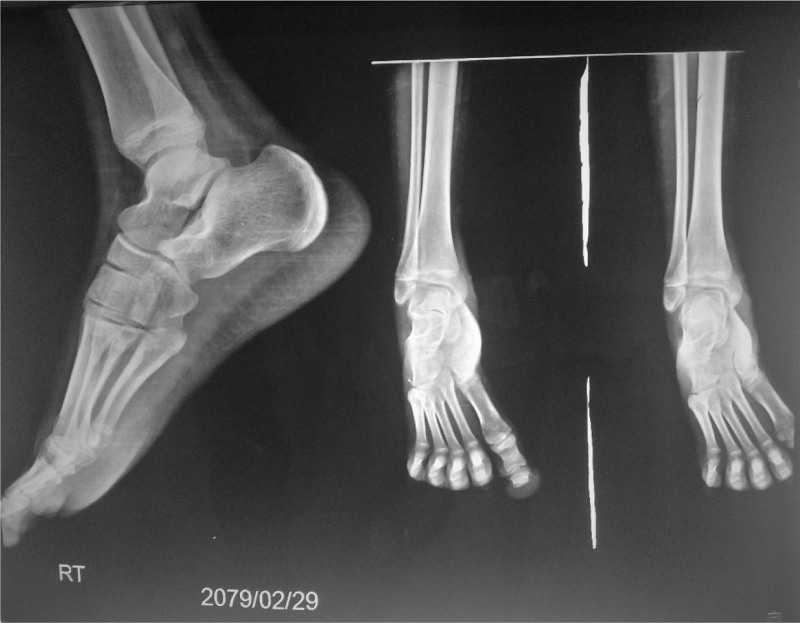
Normal lower foot X-ray.

**Figure 4.**
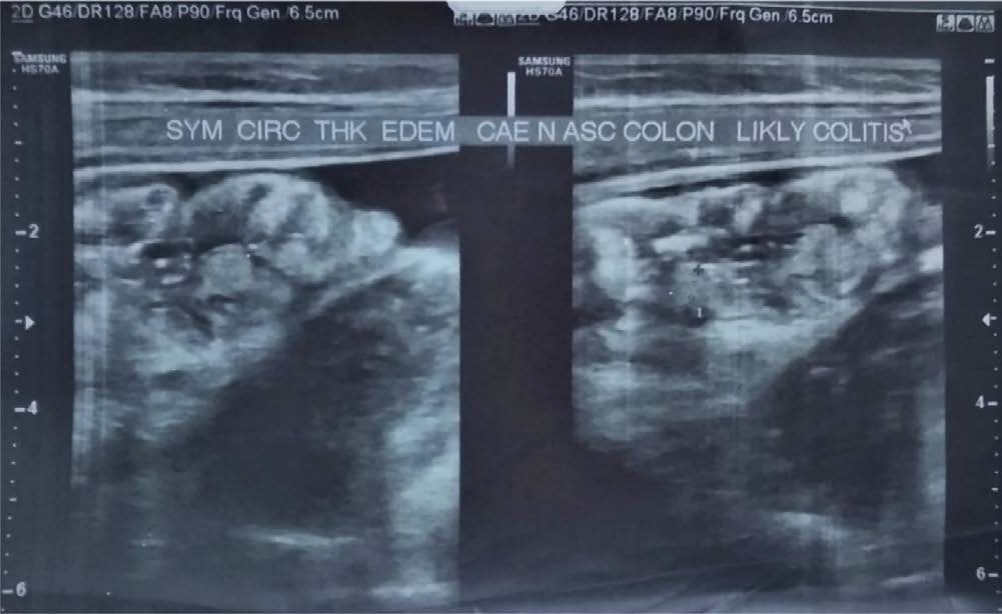
Symmetrical circumscribed thickening and edematous cecum and ascending colon.

Ophthalmologic tests were normal. Lower extremity radiography was normal (Fig. 3) and ultrasonography of the right ankle was also normal which showed thickened skin and subcutaneous tissue in the medial aspect of the right ankle. Abdominal ultrasound showed symmetrical circumscribed thickening and edematous cecum and ascending colon with likely colitis (Fig. 4).

Biopsy-proven membranous glomerulonephropathy in the presence of ANA and anti-dsDNA meant the patient was diagnosed with SLE based on the second rule of Systemic Lupus International Collaborating Clinics (SLICC) classification.^[6]^ The medial malleolus region ulcer was debrided. The patient was given intravenous metronidazole (10 mg 3 times a day), Intravenous ciprofloxacin (20mg/kg/d), trypsin-chymotrypsin (100,000 Armour Units), oral tablet prednisolone (30 mg per oral 2 times a day), oral zinc tablet (15 mg/d), calcium with calcitriol tablets oral tablets (0.5 mg/d), oral hydroxychloroquine tablets (150 mg per oral once daily) and Sunscreen having Sun Protection Factor 50% to apply at 8 am and 2 pm on face and body. The treatment was well tolerated and, therefore, continued. Patient was called after 1 month for follow-up. Currently her wound size has been reduced and dysentery has been resolved. No adverse event was reported. The number (and types) of investigations performed were limited due to financial constraints. A timeline of key events is presented in Table 1.

**Table 1:** Timeline.

Date	Events
2022-06-06	Child presented to OPD with complaint of a single superficial round ulcer over right medial malleolus of size approximately 6 × 6 cm for 2 months. Cefixime medication was given and follow up in 2–3 days was done for wound dressing but the wound did not subside.
2022-06-10	Child also complains of passes of loose stool 4 days back which was acute in onset, watery in consistency, blood and water mixed that occurred 5–7 episodes throughout the day.
2022-06-11	Blood investigations were done and showed anemia of chronic diseases. ESR was raised over 53 mm/hr. Urine routine examination and microscopy showed presence of WBCs and bacteria.
2022-06-12	Ultrasound of urinary bladder showed dependent debris on lumen with normal distension and wall thickness. Stool specimen revealed the presence of *Entamoeba* *histolytica*. ANA and anti-dsDNA tests were positive. Renal biopsy showed membranous glomerulonephropathy, class V according to WHO classification. Diagnosis of SLE was made based on SLICC criteria. The medial malleolus ulcer was debrided. The patient was given metronidazole, ciprofloxacin, trypsin-chymotrypsin, prednisolone, zinc, calcium with calcitriol tablets, hydroxychloroquine tablet, and sunscreen.
2022-07-15	The wound size was reduced and dysentery was resolved. No adverse events were reported.

ANA = antinuclear antibody, anti-dsDNA = anti-double stranded DNA, ESR = erythrocyte sedimentation rate, OPD = outpatient department, SLE = systemic lupus erythematosus, SLICC = Systemic Lupus International Collaborating Clinics, WBCs = white blood cells, WHO = World Health Organization.

## 3. Discussion

SLE is an autoimmune disease which is less common in children. About 20% are diagnosed between prior 16years.^[2]^ Its clinical course ranges from mild gradual onset to high complications leading to multi-organ failure. Due to variance in the presentation SLICC gave classification criteria.^[6]^

The involvement of gastrointestinal tract is common in SLE, however the gastrointestinal manifestations are mostly due to adverse reactions of various drugs and superimposed infections rather than due to SLE.^[7]^ Among gastrointestinal manifestations Lee et al^[8]^, reported the presence of abdominal pain in around 22% in people with SLE which was also present in this patient. Patient complained of having dysentery and leg ulcers; which has not been co-existed together in any of the other case reports till now. Even the cases of diarrhea has been reported in SLE. The case presenting with dysentery is too rare. One case report by Butt et al^[9]^, described the women with SLE on steroid therapy presentation of dysentery, fever, and thrombocytopenia and later revealed due to histoplasmosis. The cause of dysentery may be due to either vasculitis or colitis shown in the ultrasound. Stevens et al^[10]^ reported occurrence of SLE in ulcerative colitis patient. Two other reported cases with dysentery in SLE were due to amebic colitis among which one was fatal while the other one was successfully treated with metronidazole and paromomycin.^[11,12]^ Complications such as secondary bacteremia, disseminated intravascular coagulation, acute oliguric renal failure, acute respiratory distress syndrome, and intestinal perforation were reported. While our patient also presented with this rarely reported combination of SLE and dysentery in which none of these complications were observed. However, cystitis and proteinuria were seen in our case.

In the retrospective study done by Shanmugam et al, among 23% of wounds caused due to various immune diseases, only 0.05 to 0.15% had wounds due to SLE.^[4]^ In this case report, the patient had an ulcer in the right medial malleolus who was later diagnosed with SLE. Though, the exact reason was not found for not healing of the ulcer but vasculitis and Raynaud’s phenomenon can be the reason as described by Battung et al and Lederhandler et al respectively,^[13,14]^ whereas Kissin et al^[15]^ reported the use of hydralazine induced SLE can present with ulcer. Grossberg et al, reported 2 case reports on the presence of ulcerative plantar keratoderma in SLE patients.^[16]^

The specificity and sensitivity of ANA is 96.52% and 90%, respectively, whereas the specificity and sensitivity of anti-dsDNA is 97.4% and 57.3%, respectively, in patients with SLE.^[17,18]^ This patient was also positive for ANA and anti-dsDNA. A meta-analysis performed by Unterman et al^[19]^ showed the prevalence of neuropsychiatric manifestations was present in 56% among them headache, cognitive dysfunction, mood disorders, and seizures were common but these symptoms were absent in this patient. Wysenbeek et al^[20]^ reported photosensitivity in 73% patients with SLE involving more in the face than arms, chest, and trunk which was even absent in this patient.

Irrespective of patient history and presentation, all the SLE patients are advised to avoid sunlight, use sun cream, and wear cloths and hats. Corticosteroids (1–3 mg/kg/d), anti-malarial medication (hydroxychloroquine and chloroquine) 4–6 mg/kg/d along with metronidazole (35–50 mg/kg/d) has been found more effective in the treatment of SLE induced dysentery. Other options include the use of azathioprine (0.5–2.5 mg/kg/d), methotrexate (7.5–15 mg/kg/wk), cyclophosphamide (0.5–2.5 mg/kg/d). Use of tocilizumab has shown improvement in some patients with SLE.^[21]^ TNF-alpha inhibitor has been found more effective in the treatment of SLE with arthritis. Currently research are going on the use of inhibition of Janus kinases, SYK Kinases and Stat phosphorylation in treatment of SLE.^[22]^ For management of leg ulcers use of antibiotics along with wound dressing has been effective along with the treatment of SLE in this patient.

SLE is a chronic condition which is difficult to diagnose early and may present with complications. While treatment options were limited due to availability and affordability of drugs, timely diagnosis and start of proper treatment prevented further complications in this case.

## 4. Conclusion

We reported an atypical case of SLE with dysentery and chronic leg ulcer as presenting problems. Dysentery in SLE has been reported to be fatal in the past. Decreased cellular components of immunity can make an SLE patient susceptible to infections. Immunosuppressive agents, the treatment mainstay, can further add to this already significant cause of morbidity and mortality in SLE patients. Poor wound healing in SLE may also contribute by providing a breeding ground for harmful pathogens. Therefore, any suspicion of infection in SLE should be treated aggressively with antibiotics.

## Acknowledgments

The authors thank the Department of Pediatrics and Adolescent Medicine of B.P. Koirala Institute of Health Sciences, Dr Basanta Rai for their support.

## Author contributions

**Conceptualization:** Biki Kumar Sah.

**Data curation:** Biki Kumar Sah, Shipra Chaudhary, Aasha Ghimire.

**Formal analysis:** Biki Kumar Sah, Ashhrik Pahari.

**Funding acquisition:** Shipra Chaudhary, Aasha Ghimire.

**Investigation:** Aasha Ghimire, Neelam Kumari.

**Methodology:** Rajan Kumar Sah, Neelam Kumari, Ashhrik Pahari.

**Project administration:** Biki Kumar Sah, Abhishek Kumar Sah, Ashhrik Pahari.

**Resources:** Biki Kumar Sah, Shipra Chaudhary.

**Software:** Yaswant Kumar Jaiswal, Vivek Kumar Sah.

**Supervision:** Shipra Chaudhary.

**Validation:** Shipra Chaudhary, Ashhrik Pahari.

**Visualization:** Aasha Ghimire, Rajan Kumar Sah.

**Writing—original draft:** Biki Kumar Sah, Ashhrik Pahari, Shipra Chaudhary.

**Writing—review and editing:** Shipra Chaudhary, Ashhrik Pahari, Rajan Kumar Sah, Biki Kumar Sah.
